# Expression of DNA Translesion Synthesis Polymerase η in Head and Neck Squamous Cell Cancer Predicts Resistance to Gemcitabine and Cisplatin-Based Chemotherapy

**DOI:** 10.1371/journal.pone.0083978

**Published:** 2013-12-20

**Authors:** Wendi Zhou, Yih-wen Chen, Xiyong Liu, Peiguo Chu, Sofia Loria, Yafan Wang, Yun Yen, Kai-Ming Chou

**Affiliations:** 1 Department of Pathology, St. Luke’s-Roosevelt Hospital Center, Affiliated Hospital of Columbia University College of Physicians and Surgeons, New York, New York, United States of America; 2 Department of Pharmacology and Toxicology, Indiana University School of Medicine, Indianapolis, Indiana, United States of America; 3 Department of Molecular Pharmacology, Beckman Research Institute of the City of Hope National Medical Center, Duarte, California, United States of America; 4 Department of Pathology, City of Hope Comprehensive Cancer Center, Duarte, California, United States of America; Ludwig-Maximilians University, Germany

## Abstract

**Purpose:**

The development of resistance against anticancer drugs has been a persistent clinical problem for the treatment of locally advanced malignancies in the head and neck mucosal derived squamous cell carcinoma (HNSCC). Recent evidence indicates that the DNA translesion synthesis (TLS) polymerase η (Pol η; hRad30a gene) reduces the effectiveness of gemcitabine/cisplatin. The goal of this study is to examine the relationship between the expression level of Pol η and the observed resistance against these chemotherapeutic agents in HNSCC, which is currently unknown.

**Methods:**

Sixty-four mucosal derived squamous cell carcinomas of head and neck (HNSCC) from 1989 and 2007 at the City of Hope National Medical Center (Duarte, CA) were retrospectively analyzed. Pretreatment samples were immunostained with anti-Pol η antibody and the correlation between the expression level of Pol η and clinical outcomes were evaluated. Forty-nine cases treated with platinum (n=40) or gemcitabine (n=9) based chemotherapy were further examined for Pol η expression level for comparison with patient response to chemotherapy.

**Results:**

The expression of Pol η was elevated in 67% of the head and neck tumor samples. Pol η expression level was significantly higher in grade 1 to grade 2 tumors (well to moderately differentiated). The overall benefit rate (complete response+ partial response) in patients treated with platinum and gemcitabine based chemotherapy was 79.5%, where low Pol η level was significantly associated with high complete response rate (p=0.03), although not associated with overall survival. Furthermore, no significant correlation was observed between Pol η expression level with gender, age, tobacco/alcohol history, tumor stage and metastatic status.

**Conclusions:**

Our data suggest that Pol η expression may be a useful prediction marker for the effectiveness of platinum or gemcitabine based therapy for HNSCC.

## Introduction

Mucosal derived squamous cell carcinoma of the head and neck (HNSCC) refers to a group of biologically similar cancers originated from the mucosal squamous epithelial lining of upper aerodigestive tract, including the lip, oral cavity, nasal cavity, paranasal sinuses, pharynx, and larynx. HNSCC is the sixth most frequently occurring cancer worldwide and accounts for 2% of all cancer death annually. According to the American Cancer Society, 36,540 Americans were diagnosed with head and neck cancer in 2011 and 7,880 died from the disease [1,2]. Most patients present lymph node metastatic disease at the time of diagnosis, and the five-year survival rate of those patients is around 35% [3]; which has not improved over the last decade [4]. Platinum-based combination regimen, such as cisplatin/oxaliplatin plus 5-FU and taxotere, is the current first-line neoadjuvant chemotherapy for locally advanced HNSCC [22]. However, the poor or partial response to platinum-based chemotherapy of HNSCC remains an enigma for oncologists. Platinum compounds form DNA intrastrand or interstrand cross-links that severely block DNA synthesis and result in mutations and apoptosis [5]. These platinum induced adducts are repaired by nucleotide excision repair system (NER) [6,7], the mismatch repair (MMR) system, and recombination repair (RR) [8]. In addition, DNA translesion synthesis (TLS) polymerases have also been shown to have the ability to bypass cisplatin-induced intrastrand adducts [9,11,39,40]. This suggests these bypass polymerases provide an alternate mechanism in handling platinum compound induced DNA adducts and may contribute to the observed resistance against these compounds [9,11]. Among the TLS DNA polymerases, DNA Polymerase η (Pol η; hRad30a gene; xeroderma pigmentosum variant gene product) is the only one with well-understood biological function, which is to replicate across the cis-syn cyclobutane pyrimidine dimers (CPDs) that induced by UV radiation [10]. Genetic defects in the gene encoding Pol η results in Xeroderma Pigmentosum Variant (XP-V) disease, and XP-V patients are highly sensitive to UV irradiation and prone to the development of skin cancer [10]. Pol η has also been shown to have the ability to bypass a broad range of DNA lesions, such as 7,8-dihydro-8-oxoguanine [15], (+]-trans-anti-benzo[α]pyrene-N2-dG [16], acetylaminofluorene-adducted guanine [17], O6-methylguanine [18], and thymine glycol [41]. 

In addition, it has been demonstrated that Pol η can modulate the cellular sensitivity to chemotherapeutic agents [11]. The Pol η deficient cells derived from XP-V patients were more sensitive to β-D-arabinofuranosylcytosine (cytarabine, araC), gemcitabine, and cisplatin [12]. Cellular and biochemical analyses suggested that the higher sensitivity of XP-V cells to these agents is due to the lack of Pol η activity in facilitating the resumption of the paused DNA replication caused by therapeutic agents [13,14]. Pol η bypasses the Pt-GG intrastrand crosslinked adducts with a relative higher efficiency and fidelity, as comparing to other TLS enzymes [42-44]. Studies have also demonstrated that the expression level of Pol η negatively correlated with cisplatin sensitivity of non-small cell lung cancer (NSCLC) cell lines [19]. Furthermore, Pol η protein expression was suggested to be an independent predictive marker for the treatment response and survival of metastatic gastric adenocarcinoma patients who are treated with oxaliplatin-based chemotherapy [20]. 

In this study, we examined the associations between *in situ* expression of Pol η proteins and known prognostic factors including staging and tumor differentiation. We also examined the associations between expression of Pol η proteins and response to platinum or gemcitabine based chemotherapy treatment and survival in HNSCC. 

## Materials and Methods

### Patient characteristics

Tumor blocks for sixty-four patients diagnosed with mucosal derived squamous cell carcinomas of head and neck (HNSCC) from 1989 and 2007 at the City of Hope National Medical Center (Duarte, CA) were retrospectively analyzed. Tumor blocks included tumors of the nasopharynx, paranasal sinuses, oral cavity, oropharynx, hypopharynx, and larynx. The median age of the patients was 59 years (range 39–78 years) at the time of diagnosis. Eight patients were excluded for survival analysis due to the lack of long term follow-up studies. The median follow-up period was 56 months (range, 4.3–142 months). Among the 64 patients, 51 were primary HNSCC and 13 were recurrent or second primary tumor. Thirty-four patients underwent radical surgery, 49 patients received chemotherapy (40 patients received neoadjuvant cisplatin, taxotere and 5-FU, 4 patients also received gemcitabine, and the remaining 5 of the inoperable recurrent cancer patients received palliative chemotherapy (gemcitabine and hydroxyurea). Details of the clinic characteristics are summarized in Table 1. 

**Table 1:** Clinicopathological parameters and Pol η expression in 64 HNSCC.

**Characteristic**	***n***	**POLη staining**				***P ^a^***
		**Low**	**(%)**	**High**	**(%)**	
**Age (yrs)**						
**<59**	33	12	36	21	64	
**≥60**	31	9	29	22	71	0.73
**Gender**						
**Female**	17	5	29	12	71	
**Male**	47	16	34	31	66	0.34
**T stage*^b^*,*^c^***						
**T_1_**	9	3	33	6	67	
**T_2_**	10	3	30	7	70	
**T_3_**	21	7	33	14	67	
**T_4_**	17	6	35	11	65	0.56
**N status^*b*^**						
**N−**	7	2	29	5	71	
**N+**	57	19	33	38	67	0.32
**Histological differentiation**						
**Well**	8	0	0	8	100	
**Moderate**	33	7	21	26	79	
**Poor**	23	14	61	9	39	0.01
**Tobacco**						
**No**	18	7	39	11	61	
**Yes**	46	14	30	32	70	0.63
**EtOH**						
**No**	44	16	36	28	64	
**Yes**	20	5	25	15	75	0.34
**Chemotherapy**						
**No**	16	4	25	12	75	
**Yes**	48	17	37	30	63	0.2

^a^ Probability of statistical difference (P) was analyzed by χ^2^ test for independence.

^b^ Patients were classified according to the American Joint Committee on Cancer (AJCC)/International Union Against Cancer (UICC) WHO grading system and by the presence or absence of lymph node metastasis (N status).

^c^ 7 patients' T stage could not be determined.

Data represented percentages of cases with low or high Pol η expression in each parameter, and n represents absolute case number of each parameter. Probability of statistical difference (P) was analyzed by χ^2^ test for independence. Patients were classified according to the American Joint Committee on Cancer (AJCC)/International Union Against Cancer (UICC) WHO grading system and by the presence or absence of lymph node metastasis (N status).

All patients’ information and tissue sample collection were approved by the IRB committee (protocol# 95917) at City of Hope National Medical Center and the Beckman Research Institute, CA. Information regarding clinical outcome and response to chemotherapy was obtained from hospital chart review and clinical protocol record. Patients were staged according to the TNM classification system from American Joint Committee on Cancer (AJCC). Disease free survival was defined as the time from the date of confirmed diagnosis to last contact date and censored at the recurrent date. Squamous cell carcinoma was graded using Anneroth's multi-factorial grading system: G1-well differentiated, G2-moderately differentiated, G3-poorly differentiated, G4-undifferentiated. Clinical response to chemotherapy were evaluated by tumor size reduction and neck lymph node status on the computed tomography scan before and after chemotherapy, according to Response Evaluation Criteria in Solid Tumors (RECIST criteria) [21] as follows: complete response (CR)= 100% regression of the disease; partial response (PR), ≥30% decrease in the sum of the longest diameter of the targets; progressive disease≥ 20% increase; and stable disease (SD)= the remainders. Pathological complete response (pCR) was defined as absence of any gross or microscopic evidence of residual tumor in the surgical or biopsy specimen following chemotherapy. Since the pCR was only observed in four patients, therefore, only the clinical response was used for statistical analysis.

### Tissue samples and antibody

**Figure 1:**
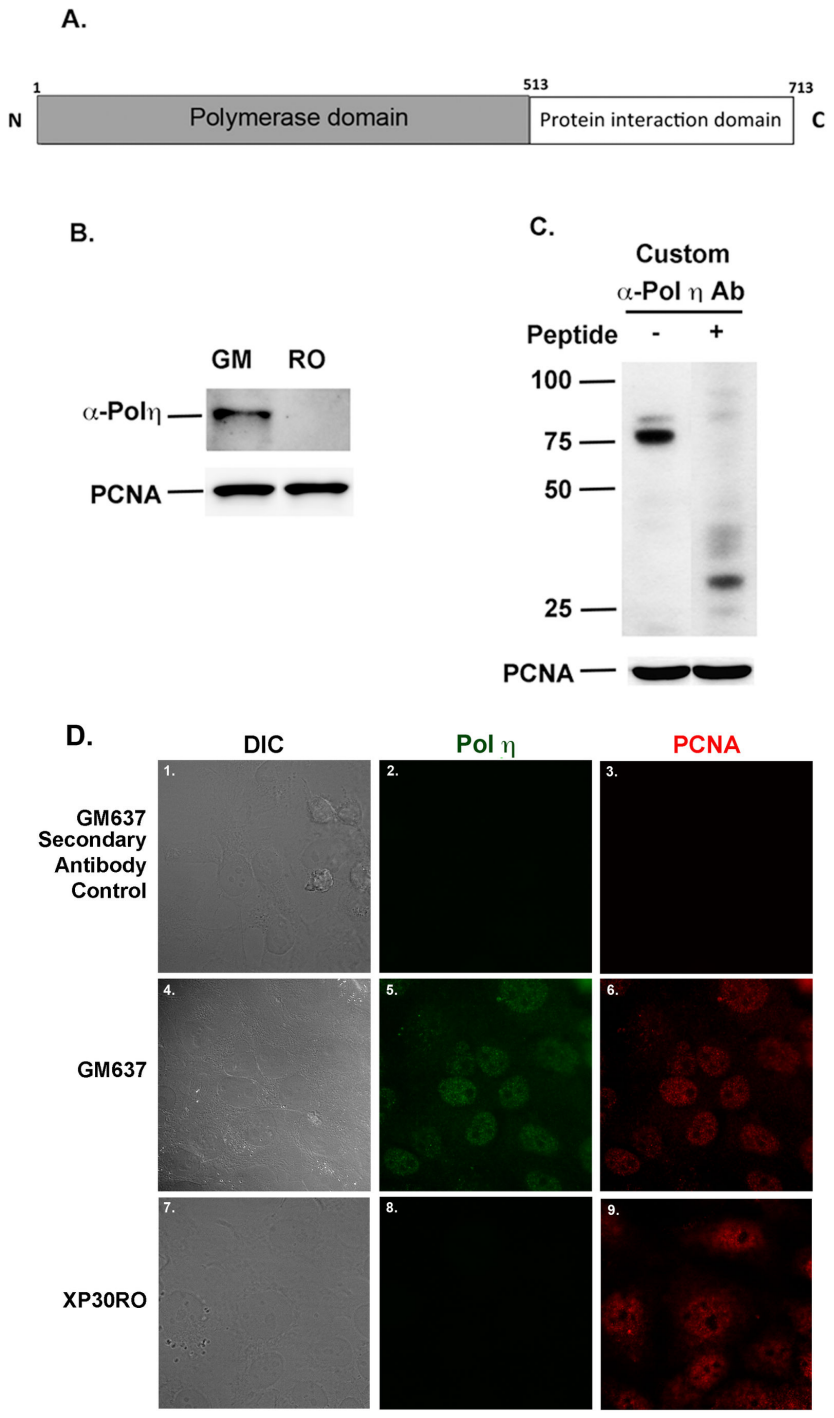
Generation and validation of anti-Pol η antibody. A) The N-terminal fragment of Pol η (highlighted in grey) used for the generation of an anti-Pol η antibody. B) The developed antibody used for the detection of Pol η in the lysates derived from GM637 or XP30RO cells. C) Lane 1. Identification of Pol η from the lysate from human HCT116 cells. Lane 2. Blocking with purified recombinant human Pol η protein. D) 1-3, GM 637 cells probed with secondary antibody alone (D2 and D3). 4-6, GM637 cells probed with anti-Pol η antibody (D5) or anti-PCNA antibody (D6). 7-9, XP30RO cells probed with anti-Pol η antibody (D8) or anti-PCNA antibody (D9).

HNSCC samples (n= 64) and paracancer normal squamous mucosal tissues prior to chemotherapy were obtained from patients undergoing operation for carcinoma resection or incisional/excisional biopsy with written informed consent of patients from January 1989 to June 2007. Nine out of sixty-four post-chemo tumor tissues were also compared. Histological grading was assessed according to the 1987 International Union against Cancer (UICC). The anti-Pol η antibody was generated by immunizing rabbit with highly purified N-terminal domain of human Pol η protein (amino acid 1 to 513, see Figure 1a). The polyclonal antibody was purified using a G protein column. Antibody specificity was evaluated with western blotting and blocking assay (Figure 1b and 1c). 

### Western blot analysis

Western blot was conducted as previously described [1]. Briefly, 20-μg of total cell lysate was separated by 10% SDS-PAGE before transferred to a nitrocellulose membrane (Bio-Rad Laboratories, CA) and incubated with blocking buffer (1% I-Block reagent and 0.1% Tween 20) containing Pol η primary antibody overnight at 4°C. The membrane was washed with PBS with 0.1% Tween 20 for 3-5 times before incubated with fluorescent-labeled secondary antibodies for additional 60 minutes. After sequential washes, the signals on the membranes were scanned with an Odyssey Infrared Imaging System (LI-COR Biosciences) or detected using a Fuji LAS-1000 imaging system. 

### Immunohistochemistry

Human GM637 wild type and the Pol η deficient XP30RO cells were used to validate the antibody developed in the laboratory. Briefly, the proliferative cells were fixed with 4% paraformahyde in PBS followed by incubation with permeabilization buffer (0.5% saponin, 0.1% BSA and 0.1% NaN3 in PBS) at room temperature for 30 min before the addition of primary antibodies to the permeabilization buffer for additional 1 h at room temperature. The primary antibodies were removed by washing the cells three times with permeabilization buffer before the addition of DyLight 488 or DyLight 549 conjugated secondary antibodies (Jackson ImmunoResearch Laboratories, Inc.) for additional 1 h. The cells were examined using confocal microscopy (Biorad). 

For the expression of Pol η in paraffin-embedded tissue blocks obtained from patients, immunohistochemical staining was performed using 4-μm thick sections prepared from formalin fixed blocks. Tissue sections were deparaffinized in xylene followed by 100% ethanol. Samples were then quenched in 3% hydrogen peroxide and pretreated to promote antigen retrieval by steaming with EDTA solution. After antigen retrieval, slides were incubated in Protein Block (DAKO, Carpinteria, CA) for 5 minutes followed by incubation with primary anti-Pol η antibodies for 30 minutes at room temperature. The slides were then washed in Dako buffer and incubate with DAKO Envision with anti-rabbit Polymer secondary antibody (DAKO, Carpinteria, CA) for additional 30 minutes. After washes in Dako buffer, slides were incubated with the chromogen diaminobenzidine tetrahydrochloride (DAB, Sigma-Aldrich, St. Louis, MO), counterstained with hematoxylin, and mounted.

The staining of Pol η was evaluated by counting of positive nuclear stained cells. In general, the staining of Pol η was crisp, with minimal background staining of paratumor stromal cells as shown in Figure 1. A total of 20 high power fields were counted for each samples, and the percentage of positive tumor cell nuclei was counted for each case (magnification ×400, field size 0.18 mm^2^) in areas with most positive nuclei (hot spots). Nuclear immunoreactivity was scored based on the staining intensity and staining extent, and was graded using a two tier grading system: low expression: nuclear staining in <30% tumor cells; and high expression: nuclear staining in >30% tumor cells. All cases were scored blindly by three observers and showed an inter-rater reliability (intra-class Correlation Coefficient, Cronbach's α = 0.82). 

### Statistical Analysis

The relationship between Pol η expression and clinicopathological characteristics was examined by a chi-square test and Fisher's exact tests. Univariate analysis of disease free survival was done by Kaplan-Meier analysis (log-rank test). Multivariate analysis was carried out with Cox's proportional hazard model adjusting for major clinicopathologic parameters. The P values (two-sided) less than 0.05 were considered as statistical significance. All data was analyzed using the JMP statistical software, Version 9 (Cary, NC). 

## Results

### Validation of anti-Pol η antibody

The anti-Pol η antibody was developed by immunizing the rabbit with highly purified human Pol η polymerase domain within the N-terminal (1-513 amino acids, Figure 1a), the detailed procedures were described in the Materials and Methods. To test the specificity of the developed antibody, we used this antibody to probe the presence of Pol η using cell lysates from the human fibroblast GM637 cells or the XP30RO fibroblast cells derived from XP-V patients. As shown in Figure 1b, the antibody recognized a ~80 kDa protein from the GM637 cell lysates that matches with the molecular weight of Pol η but the same protein band was not detected in the XP30RO cell lysates. To further this result, a competition assay using a highly purified N-terminal fragment of Pol η to compete with the endogenous cellular Pol η was also performed. As shown in Figure 1c, the anti-Pol η antibody specifically recognized a dominant protein band at ~80 kDa, which is consistent with the molecular weight of Pol η. In the competition assay (Figure 1c, lane 2), this recognized protein band was completely blocked by the purified human Pol η, indicating the developed anti-Pol η antibody has a high specificity to recognize human Pol η. To further confirm the specificity of this antibody, we performed an IHC study using wild type human GM637 and XP30RO fibroblast cells derived from a XP-V patient. As shown in Figure 1D 5, the anti-Pol η antibody recognized Pol η in the nuclei of GM637 cells but not in the Pol η deficient XP30RO cells (Figure 1D 8). As internal control, anti- proliferating cell nuclear protein (PCNA) signals were positive in both cells (Figure 1D. 6 and 9). No signals were detected when only secondary antibody was present (Figure 1D 2-3). These results further confirmed the specificity of anti- Pol η antibody.

### Expression of Pol η was positively associated with differentiation in HNSCC

The relationship between clinicopathological parameters and Pol η expression was investigated and the results were listed in Table 1. A significant correlation was observed between the expression of Pol η and histological grade in HNSCC (p <0.01). In addition, the percentages of Pol η-high expression level was found to be correlated with the differentiation of HNSCC from poor, moderated to well, as the percentages of Pol η-high expression level were 39, 79, and 100%, respectively. Other clinicopathological parameters including age of patients at diagnosis, gender, T stage, N stage and tobacco/alcohol history were also evaluated, but no statistical significance was observed. 

### Immunostaining of Pol η in normal squamous epithelial tissue and HNSCC

**Figure 2:**
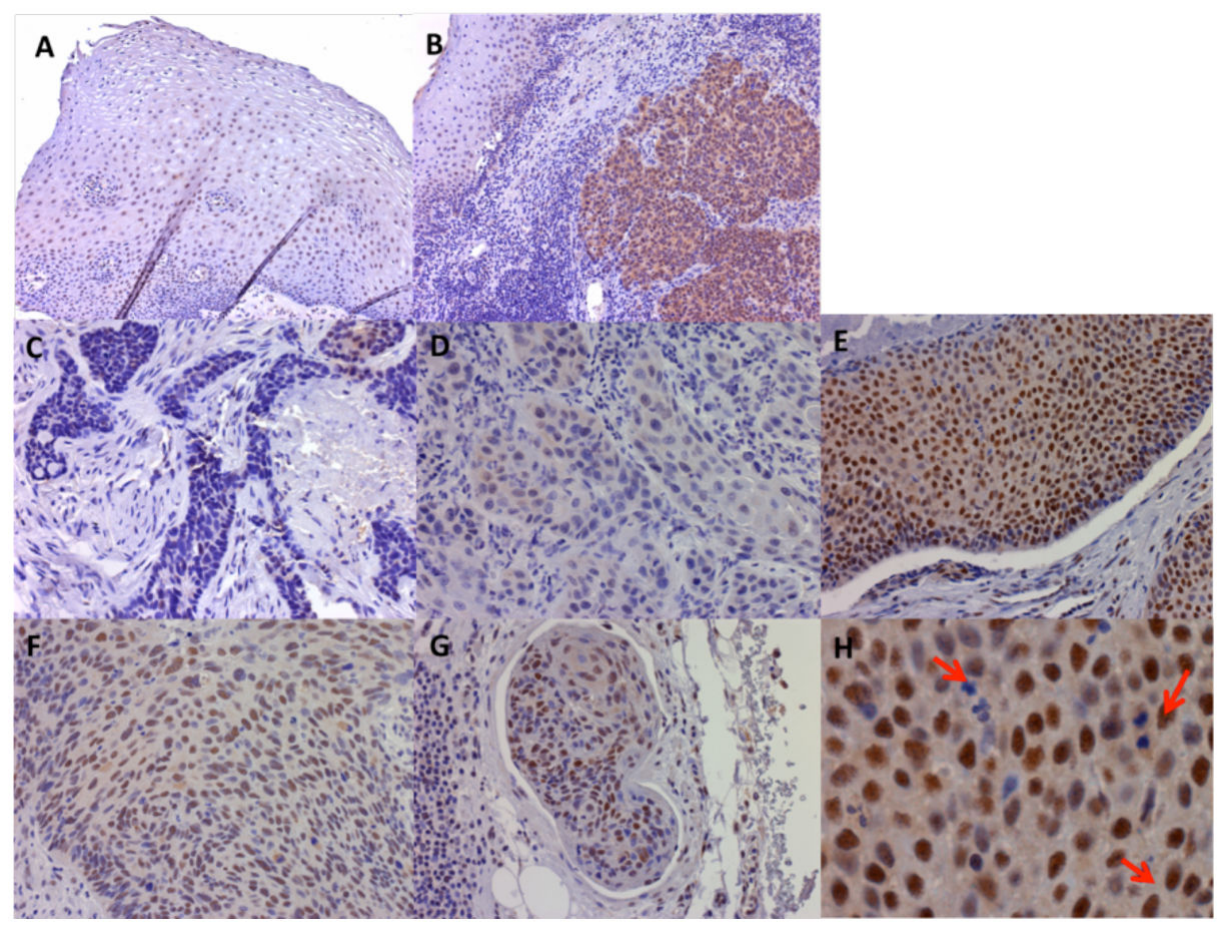
The expression of Pol η protein in head and neck squamous cell cancer. A) Nuclear staining of Pol η in normal squamous epithelium as control. B) Comparison of Pol η staining level between HNSCC and para-cancer normal squamous mucosa in oral cavity. C-E) HNSCC nuclear Pol η expression was classified as negative (C), low (D) and high, according to the frequency and intensity of the stained cells. F-G) Comparison of Pol η staining in primary cancer in soft palate (F) and metastatic cancer cells to lymph node (G). (Immunoperoxidase, original magnification: ×100 (A-B), ×200 (C-G)).

The Pol η expression level in HNSCC tissue was compared to the noncancerous mucosal squamous epithelium (Figure 2). As shown, Pol η is present in the basal and parabasal layers of the normal squamous epithelium. In the interstitial tissue, only trace amount of Pol η was detected in the stromal cells. A high Pol η expression was observed in the germinal centers, which is consistent with previous studies and its role in somatic hypermutation in germinal center [45-47]. In comparison with the normal squamous epithelium, an increased level of Pol η expression was observed in the head and neck squamous cell carcinomas as 43 of 64 carcinomas (67%) samples showed positive stain. Squamous carcinoma cellular differentiation is a characteristically most discernable as the presence or absence of keratinization. Keratinization takes place in the form of keratin pearls and is gathered toward the center of carcinoma cell nests. The carcinoma cells in the center often exhibit an increased differentiation phenotype than the edges of the nests [55]. Although Pol η staining was higher in well-differentiated carcinoma, no preferential localization of Pol η in the tumor nests was observed [23]. The expression level of Pol η in carcinoma cells that metastasized to the lymph nodes was also examined and similar expression level was observed as comparing to the corresponding primary sites (Figure 2). Another interesting observation was that the mitotic cancer cells showed low Pol η expression, which was consistent with the fact that Pol η is mainly required during G1/S phase [48-50].

### Alteration of Pol η protein levels in response to platinum based-chemotherapy

**Figure 3:**
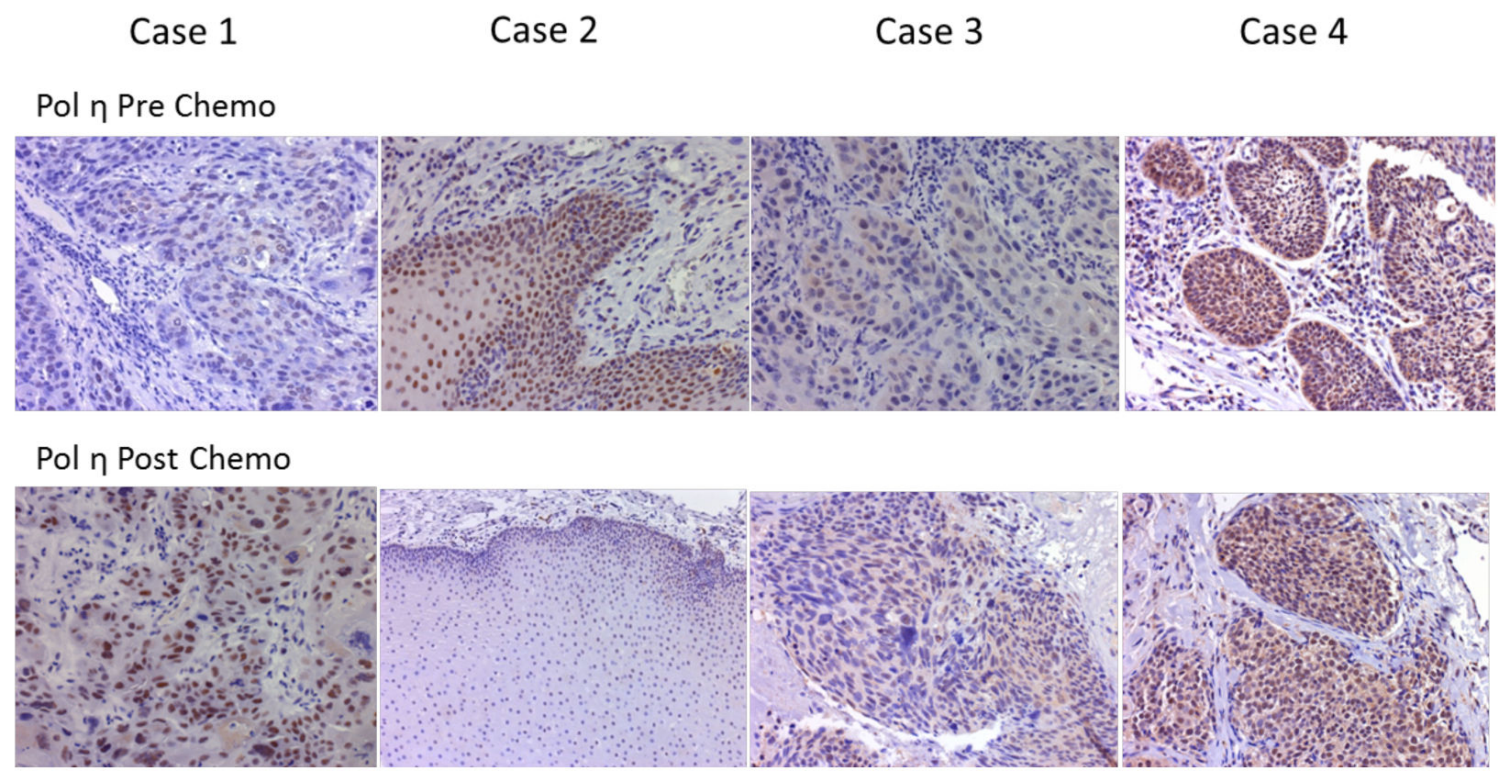
Platinum-based chemotherapy and Pol η protein expression in head and neck squamous cell cancer. Upper row represents nuclear staining of Pol η in HNSCC before chemotherapy. Lower row represents Pol η staining in tissue obtained from the same areas of original tumors after chemotherapy. Case 1) Pol η staining level up-regulated post chemotherapy. Case 2) Pol η staining level down-regulated post treatment. Case 3-4) Pol η staining level remained same intensity post treatment (immunoperoxidase, original magnification x200).

To determine whether platinum based chemotherapy altered Pol η expression level, samples from the same HNSCC patients pre- and post-chemotherapy were analyzed and compared (n=9, Figure 3 and Table 2). We found 6/9 HNSCC (67%) had similar or increased expression of Pol η after chemotherapy (including one case that was nearly negative in Pol η expression before chemotherapy) and 3/9 (33%) have decreased Pol η expression after chemotherapy. The effect of chemotherapy was evaluated by both tumor size reduction in radiographical examination and histological examination of the removed carcinoma tissue post treatment. The outcome of chemotherapy was categorized as complete remission (CR), partial response (PD), stable disease (SD) and progression disease (PR). Data indicated that 33% CR (2 cases), 50% PR (3 cases) and 17% PD/SD (1 case) showed similar up-regulated Pol η expression post chemotherapy, and 33% CR (1 case), 67% PR (2 cases) and 0% PD showed decreased Pol η expression. However, no significant correlation between the expression change of Pol η in response to chemotherapy, mainly due to the small sample size. 

**Table 2:** Regulation of Pol η protein expression level post chemotherapy.

	**CR ^*a*^**	**(%)**	**PR ^*a*^**	**(%)**	**PD+SD ^*a*^**	**(%)**	**n**	**P**
**up-regulation of Pol η**	2	33	3	50	1	17	6	
**down-regulation of Pol η**	1	33	2	67	0	0	3	N/A

^a^ CR, complete response; PR, partial response; SD, stable disease; PD, progression disease.

### Clinical Significance of Pol η Staining Level in HNSCC

Previous studies have indicated that the basal Pol η expression before treatment may affect the outcome of platinum compounds based chemotherapy [19,20]. Therefore, the association between the expression of Pol η before treatment and clinical outcomes including chemotherapy response and patient survival were examined. As shown in Table 3, in the 12 subjects who achieved complete remission (CR), 8 subjects (66%) had negative/low expression of Pol η, and only 4 subjects (33%) showed high Pol η staining. In the contrary, in the 10 subjects who failed to respond (PD+SD), 8 were from high Pol η staining group (80%), only 2 subjects had low Pol η staining (20%). 

**Table 3:** Association between Pol η protein expression and clinical response to chemotherapy.

	**Cases**	**Pol η low (%)**	**Pol η high (%)**	**P value**
**CR ^*a*^**	12	8 (66)	4 (33)	
**PR**	27	8 (30)	19 (70)	
**PD+SD**	10	2 (20)	8 (80)	0.03

^a^ CR, complete response; PR, partial response; SD, stable disease; PD, progression disease. Details are explained in M&M.

**Figure 4:**
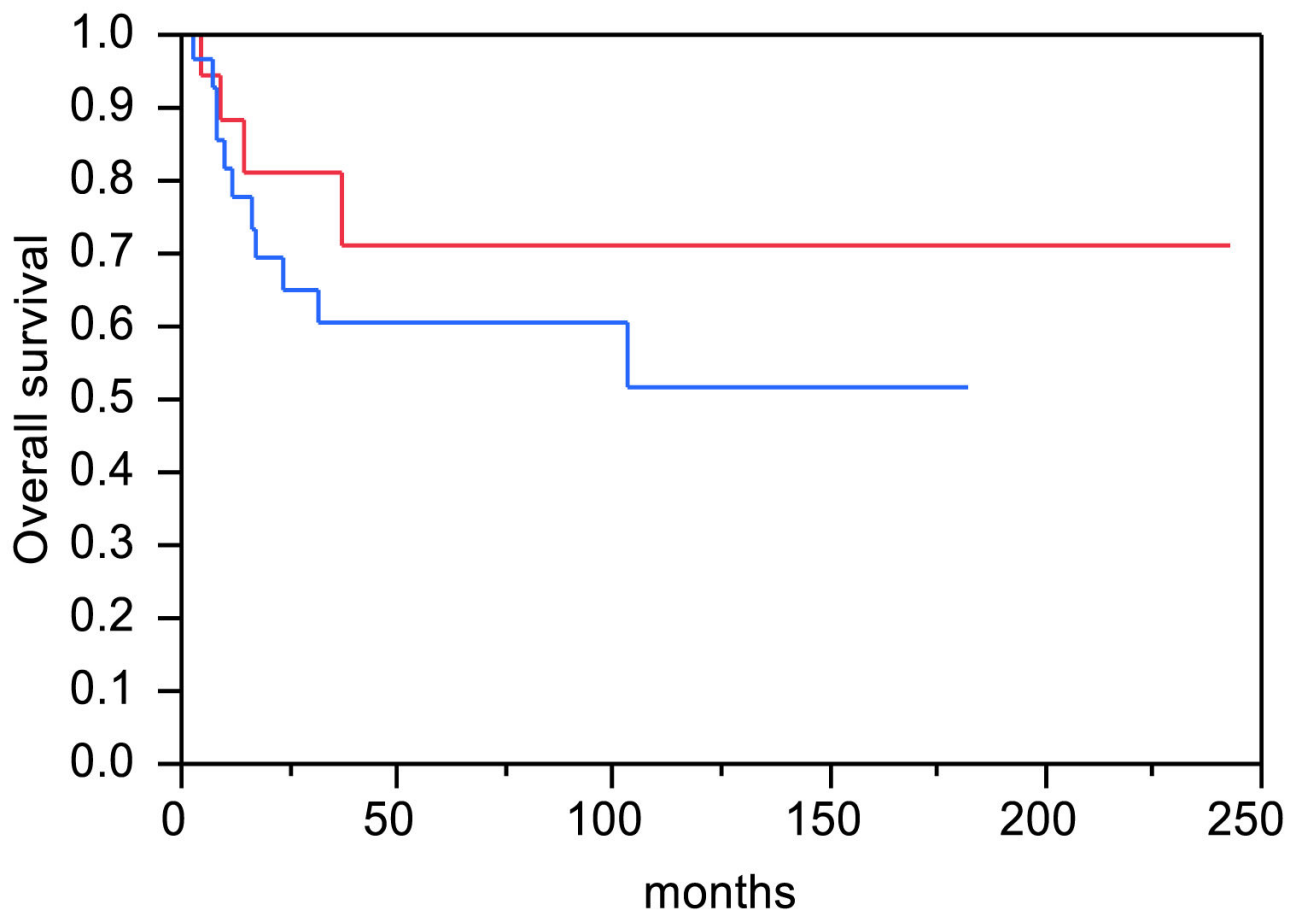
Kaplan-Meier survival probability comparing tumors with high Pol η expression to those with low Pol η expression. The blue curve corresponds to patients with high Pol η level and the red curve to patients with low Pol η level.

The relationship between disease specific survival and the expression level of Pol η was further analyzed using Kaplan-Meier analysis (log-rank test). Although there is a trend showing that patients with high Pol η expression tend to have shorter survival, the correlation was not significant (Figure 4). Multivariate analysis was further performed with Cox's proportional hazard model adjusting for major clinicopathologic parameters. As shown in Table 4, T stage (P= 0.03) was the only parameter found to be a significant independent predictor of death from carcinomas. No other variables including Pol η staining, age, N status, and histological tumor differentiation were associated with survival according to the multivariate analysis. The lack of association between Pol η expression and patient survival in our study could be due to the small sample size collected over a very long period. High local recurrence rate is a well-known factor precluding long-term tumor-free survival of the patients with HNSCC [2-4,22]. In our cohort, there were 23 out of 64 patients died of disease recurrence, including 16 patients with high Pol η level and 7 patients with low Pol η level. However, no statistical significance was found between Pol η expression and cancer recurrence (p= 0.15, data not shown). 

**Table 4:** Contribution of various potential prognostic factors to disease-free survival in oral carcinoma patients (n = 58).

**Parameters**	**Risk ratio**	**95% CI^*a*^**	***P^b^***
**Age**	2.71	0.85-10.22	0.09
**T stage**	3.47	2.17-6.98	0.03
**N status**	1.04	0-4.39	0.23
**Histological differentiation**	2.98	0.72-14.8	0.12
**POL η staining**	0.9	0.20-3.92	0.86
**chemotherapy**	0.79	0.08-17.62	0.85
**Tobacco**	2.24	0.54-11.4	0.32
**EtOH**	1.42	0.61-3.39	0.26

^a^ CI, confidence interval.

^b^ P value was obtained by Cox proportional hazards method. Calculated by log-rank test.

## Discussion

It is known that platinum-based anticancer agents inhibit tumor growth mainly by forming DNA adducts [50]. The high expression level of Pol η has been correlated with poor clinical outcome when platinum-based agents were used for the management of non-small-cell lung cancer and metastatic gastric adenocarcinoma [19,20]. According to the Human protein atlas project, Pol η is ubiquitously expressed in nuclei in essentially most tissues except the mesenchymal-, glial- and stromal cells in endocrine tissues [28]. In addition, Pol η has been observed in most carcinoma cells, although different expression levels have been reported [19,20,28,29]. Therefore, the potential impact of Pol η on the activity of platinum-based drugs or gemcitabine in HNSCC is examined in this report. 

In this study, we observed that Pol η was mainly expressed in the highly proliferating basal and parabasal layers of normal mucosal squamous epithelium. A much higher expression level of Pol η expression was detected in the majority of HNSCC as compared to the normal counterpart tissues. In addition, a higher Pol η expression level in well-differentiated HNSCC was detected as compared to poorly-differentiated ones (Figure 2 and Table 1). We also observed that high basal level of Pol η expression before chemotherapy is significantly correlated to the low response rate to chemotherapy. In previous studies, it has been shown that Pol η reduces the cytotoxic effects of both gemcitabine and cisplatin by extending DNA with gemcitabine at the 3’ termini [11] or bypassing cisplatin-induced intrastrand adducts [13]. In our cohort, only 5 patients received gemcitabine single treatment and 4 patients received both cisplatin and gemcitabine treatments. We combined these 9 cases with 40 cases treated with cisplatin for analysis. Patients with low basal expression of Pol η had a higher rate of complete response (8/18) than those who have high basal Pol η expression (4/31) (p=0.03) (Table 3). Our data further supported that bypass activity of Pol η affects platinum/gemcitabine activity in the management of HSCCC, and the expression level of Pol η may be useful for the prediction of a clinic response before chemotherapy. The overall benefit rate (CR+PR) was not significantly different in these two groups of patients (16/18 in Pol η low group and 23/31 in Pol η high group) (data not shown), which mainly was due to high partial response rate (18/31) in Pol η high group. This could be due to the activity of other DNA repair mechanisms contributing to the overall sensitivity to chemotherapeutic agents in tumor tissues [24-27]. For examples, DNA nuclear excision repair proteins and BRCA1 were reported to contribute to resistance against platinum-based agents [35-38,51-54]. 

Previous studies have demonstrated that cisplatin treatment elevated expression of DNA repair genes and contributed to the observed resistance [30-34]. Ceppi et. al. reported that Pol η mRNA levels in non-small cell lung cancer (NSCLC) cell lines were significantly induced by cisplatin [19]. A previous cellular study has shown that the downregulation of DNA lesion bypass polymerase ζ (Pol ζ) in the head and neck squamous carcinoma cells sensitizes cells to cisplatin, and inhibition of hRev3 gene expression was suggested to be a potential clinical strategy to reducing resistance against cisplatin in HNSCC [56]. In this study, we observed that the expression level of Pol η level increased in 6 cases (6/9) of the 9 patients who were resistant to platinum or gemcitabine based chemotherapy, which has not been reported before. Further studies will be necessary with a larger sample size to conclude whether the alteration of Pol η expression before and after chemotherapy could be used as a predictor for development of resistance to platinum or gemcitabine based chemotherapy. 

Our current study indicated that a high level expression of Pol η is associated with a reduced sensitivity to platinum/gemcitabine treatment, suggesting that expression level of Pol η can be used as a predictive marker. Our current results also indicate that there is no significant association between Pol η expression and age, gender, primary tumor size, LN metastatic status on the basis of such cases number. Although our results did not find a significant association between Pol η expression level and patient survival (OS and PFS), there is a trend showing that higher Pol η expression is correlated with shorter survival. There are several limitations to our study. First, the Pol η staining was done on selected blocks, which might not represent the status of the entire tumor tissue. Head and neck squamous cell carcinoma is well known for its tumor heterogeneity [57]. Indeed, we did find that Pol η expression varied within same tumor. Second, our current study was based on a retrospective analysis on a relatively small size of samples over a long period (1989-2007). During the study period, advances in treatment and supportive care could have impact on the survival outcomes, thus affected the statistical significance in our study. A well-designed prospective study with extensive sampling is needed to validate the results with more homogeneous and a larger number of patients.

In conclusion, the findings of the present study indicate that immunohistochemical staining may be a useful tool in evaluating the expression level of Pol η. Our results also suggested the potential for using the basal Pol η expression level in HNSCC as a predictive marker for platinum efficacy.
